# Inconsistent Effects of Experience on Running Biomechanics May be Influenced by Study Heterogeneity and Classification Criteria: a Systematic Review and Proposal of a Revised Taxonomy

**DOI:** 10.1186/s40798-025-00870-5

**Published:** 2025-06-08

**Authors:** Rodrigo Rabello, Gauri A. Desai, Allison H. Gruber

**Affiliations:** 1 HH Morris Human Performance Laboratories, Department of Kinesiology, School of Public Health–Bloomington, Indiana University, Bloomington, IN USA; 2 Department of Biomedical Sciences for Health, Università Degli Studi Di Milano, Milan, Italy; 3 Sports and Exercise Medicine, Queen Mary University of London, London, UK; 4 Department of Kinesiology, School of Public Health, University of Maryland College Park, College Park, Maryland USA

**Keywords:** Running experience, Running gait, Running injury

## Abstract

**Background:**

Less-experienced runners are proposed to sustain more running related injuries (RRIs) than more-experienced runners because of differences in their gait biomechanics. However, the effects of running experience on biomechanics remain inconclusive. The objective of this systematic review was to examine the evidence concerning the influence of experience on running biomechanics and summarize the criteria used to classify running experience. A classification procedure for running experience was proposed based on the results.

**Methods:**

Five common databases were searched for relevant articles following PRISMA guidelines (PROSPERO_ID CRD42022296734) and the PICO framework. Peer-reviewed research reporting a statistical effect of running experience on running gait biomechanics in adults (18–65 years) were included. Exclusion criteria were: subjects with current pathologies or symptomatic injuries; reporting running only barefoot, in minimalist shoes, during sprinting, or incline/decline running; classified experience only through performance-related measures; or did not specify running experience group definition. Risk of bias was assessed with the Downs and Black checklist. Extracted data were organized in tables and synthesized descriptively due to study heterogeneity.

**Results:**

Twenty-eight studies with 916 total subjects were included. Although most studies found significance in their comparisons, no studies comparing similar gait variables found the same statistical result. Some variables compared between experience levels were examined in only one study. Experience classification criteria were inconsistent between studies; cut-offs for more-experienced ranged between 2 and 10 years and/or 15–50 km/week and cut-offs for less-experienced ranged between 0.5 and 3 years and/or 0–20 km/week. Meta-analysis was not possible due to heterogeneity among the included studies.

**Conclusion:**

Effects of experience on running mechanics were inconsistent in the current literature. The lack of consistent findings may be due to the heterogeneous criteria used to classify runners into experience groups and the inconsistency of the variables investigated. Replication studies, heterogeneous study design, and longitudinal studies are needed to determine if or how running biomechanics change as runners gain experience. Heterogeneous study designs must begin with standard experience classification criteria for the effect of experience on running biomechanics to be identified. We propose an updated taxonomy to classify runners into groups considering three facets: exposure, performance, and intention.

*Trial registration*: PROSPERO ID CRD42022296734. Registered 28 September 2022—Retrospectively registered, https://www.chictr.org.cn/bin/project/edit?pid=149714.

**Supplementary Information:**

The online version contains supplementary material available at 10.1186/s40798-025-00870-5.

## Key Points

- Current research has not consistently found statistically significant biomechanical differences between runners of different levels of experience.
- Heterogeneity in group classification and choice of biomechanical outcome variables likely explains the lack of differences.
- A new, standardized taxonomy considering three different facets of experience (exposure, performance, and intention) may help future literature synthesis.

**Supplementary Information:**

The online version contains supplementary material available at 10.1186/s40798-025-00870-5.

## Background

Running is an activity that provides many health benefits, but running-related injuries (RRI) can be a critical barrier to continued participation [1]. Less-experienced runners (also known as novices) develop a greater number of RRIs per 1000 h of running than more-experienced runners [2]. Potentially, this difference in RRI rate may be due to more-experienced runners having a greater tolerance for repetitive loads due to training-related musculoskeletal adaptations, or training regimens that incorporate more optimal work/rest regimens than less-experienced runners [3], and thus more-experienced runners are able to complete greater training volumes than novices or have reduced RRI risk completing similar training volumes as novices. Additionally, given that the task of running can be accomplished using a variety of coordinated movement patterns [4, 5], novice runners may select an injurious movement pattern (i.e., gait kinetics, kinematics, and spatiotemporal parameters) that increases the risk of RRI compared with more experienced runners, even if running volume is matched.

There is currently no consensus as to which biomechanical variables increase RRI risk [6–10], but several variables have been prospectively or retrospectively associated with different RRIs to varying evidence levels in meta-analyses. For example, greater contralateral pelvic drop was moderately associated with medial tibial stress syndrome, greater instantaneous vertical loading rate was moderately associated with plantar fasciitis, and longer ground contact time was moderately associated with patellofemoral pain syndrome [6]. Identifying if these or other potentially injurious gait mechanics differ between runners of different experience levels may reveal the underlying reasons for elevated RRI risk among novice runners.

One of the challenges comparing gait between more-experienced and less-experienced runners is the heterogeneity of definitions used to classify running experience across studies. One such inconsistency is different training volumes used to classify the same group. For example, Nielsen et al. [11] defined “novices” as individuals who ran less than 10 km total in the past year, while Koblbauer et al. [12] defined novices as individuals that run less than 2–3 times a week for 10 km and/or 45 min per session. Another inconsistency is the terms used to describe experience groups. For example, the terms used for more-experienced runners include “experienced” [13–15], “recreational” [16–18], or “competitive” [19, 20]. Furthermore, studies are inconsistent regarding the training volume that differentiates between novices and experts which can result in individuals with the same training behaviors being classified as “experts” in one study but “novice” in another. For example, novice participants in Agresta et al. [21] had 1.9 ± 0.8 years of experience and ran 32.5 ± 14.6 km/week. Participants in the upper range of 3 years (based on inclusion criteria) and 60 km/week (estimate of plus two standard deviations) would be eligible for the “experts” group in Mohler et al. [22] and in Fadillioglu et al. [23]. Therefore, the lack of standard criteria for each experience level may influence the ability to critically evaluate and synthesize the evidence regarding gait differences and RRI rates among running experience groups.

Due to inconsistent evidence regarding biomechanical differences between runners of different experience levels and the lack of consensus regarding experience level definitions, the purposes of this investigation were to systematically review the literature investigating the influence of experience on running mechanics and to summarize the criteria presently used in the literature to classify runners into different experience levels. Informed by the classifications used across the literature (e.g., volume, years, performance) and motivations for running, we sought to propose a procedure for classifying running experience.

## Methods

The protocol for this review was prospectively registered in the International Prospective Register of Systematic Reviews (PROSPERO; ID CRD42022296734) and follows the recommendations from the Preferred Reporting Items for Systematic Reviews and Meta-Analyses 2020 (PRISMA). Database searches were initially performed on December 2nd, 2021, in the following databases: PubMed, Embase, Web of Science, CINAHL and SPORTDiscus. New searches were performed on October 23rd, 2022, June 30th, 2023 and December 12th, 2023 to verify if new articles fitting the inclusion criteria had been published during the time-course of manuscript preparation.

### Search Strategy

The PICO tool was used to develop the search strategy, where the *Population* of interest was runners and non-runners, the *Intervention* was running, the *Comparison* was experience level, and the *Outcomes* were kinematics, kinetics, and spatiotemporal measurements. Titles and abstracts were searched using three sets of keywords (Population and Intervention required the same keyword), with each set being combined with the Boolean operator “AND”, and keywords within sets were separated using “OR”. The keywords used for the search are presented in Table 1 and the full search strategy for the different databases is listed in Supplementary Material 1.

**Table 1:** Search strategy adopted

Population/Intervention	Runn*
Comparisons	**AND**
	Experience* OR Novice OR Beginner OR Recreational OR Competitive OR Skill*
Outcomes	**AND**
	Biomechanic* OR Kinetic* OR Spatiotemporal OR Kinematic* OR Coordination OR Stride OR Step

### Inclusion and Exclusion Criteria

To be included in this review, studies had to be original research, peer-reviewed, written in English, and have reported a statistical effect (e.g., group comparison, correlations, or regressions) of running experience on kinematic, kinetic, or spatiotemporal measures during regular, level surface endurance running (i.e., excluding sprinting, incline/decline running, and triathlon). There were no limitations regarding running surface (e.g., overground or treadmill) or measurement tool. Studies were excluded if participants were children or adolescents (< 18 years), older adults (> 65 years), or the population of interest had current pathologies or symptomatic injuries. Studies that involved only running barefoot or in minimalist shoes, or different modes of running were also excluded (see above). Finally, studies were excluded if the definition of running experience groups was not specified or group classification depended solely on a performance-related measure (e.g., time in a half-marathon, participating in international, national, or regional competitions) given that both high and low performing runners could have the same number of years of running experience.

### Selection Process

After the removal of duplicates, two reviewers (RR and GAD) independently screened the titles, abstracts, and full-texts for inclusion. The reviewers reconvened to compare screening results following each of these stated steps. Studies were included or excluded only if there was a consensus between both reviewers. A third reviewer (AHG) was consulted when a consensus could not be reached. Reference lists from the included studies were hand-searched for qualifying studies. When found, relevant records were screened following the inclusion/exclusion criteria and selection process. The reasons for exclusion at the full-text stage were recorded. Studies meeting all inclusion and no exclusion criteria were included in this review.

### Risk of Bias and Methodological Quality Assessment

Risk of bias and overall methodological quality assessment was conducted using a modified version of the Downs and Black checklist [24]. Table 2 shows the included items. Each question was worth one point, except for question 5, which was worth 2 points, for a total of 14 total possible points. Question 5 considers possible confounders, for which the selection varies among systematic reviews. The following were selected as possible confounders based on prior literature identifying factors that influence running gait: sex [25–27], speed [28, 29], age [5, 25, 30, 31], shoe [25], foot strike pattern (except for studies that measured only spatiotemporal variables as they are not consistently affected by foot strike) [32], and running surface [29]. Studies that reported five or six confounders received 2 points, those that reported three or four received 1 point, and those that reported zero, one, or two confounders received no points. Studies were assessed independently by two reviewers (RR and GAD) and discrepancy was resolved by consensus or by consulting the third reviewer (AHG). Studies that scored 10 or more points were considered to have high methodological quality, while those that scored 9 points or less were considered to have low methodological quality [9].

**Table 2:** Methodological quality assessment for the included studies performed with a modified Downs and Black checklist [24]

Study	1	2	3	5	6	7	10	11	16	18	20	25	27	Total	%
Agresta et al. [3]	1	1	0	1	1	1	1	1	1	1	0	1	0	10	71.4
Agresta et al. [21]	1	1	0	1	1	1	1	0	1	1	1	0	0	9	64.3
Boey et al. [16]	1	1	1	1	1	1	1	0	1	1	1	0	0	10	71.4
Boyer et al. [26]	1	1	1	2	1	1	1	0	1	1	1	1	0	12	85.7
Brahms et al. [17]	1	1	0	1	1	1	1	0	1	1	1	0	0	9	64.3
Carter et al. [39]	1	1	1	1	1	1	1	0	1	1	1	0	0	11	78.6
Fadillioglu et al. [23]	1	1	1	1	1	1	1	1	1	1	1	0	0	11	78.6
Floría et al. [47]	1	1	1	2	1	1	0	0	1	1	1	0	0	10	71.4
Frank et al. [40]	1	1	1	1	1	1	1	0	1	1	1	0	0	10	71.4
Gómez-Molina et al. [33]	1	1	0	1	1	1	1	0	1	1	1	0	0	9	64.3
Hafer et al. [35]	1	1	1	2	1	1	1	1	1	1	1	1	0	13	92.9
Harrison et al. [18]	1	1	1	2	1	1	1	0	1	1	1	1	0	12	85.7
Hreljac [36]	1	1	1	2	1	1	0	0	1	1	1	1	0	11	78.6
Jiang et al. [45]	1	1	1	2	1	1	1	0	1	1	1	0	1	12	85.7
Lees and Bouracier [41]	1	1	0	1	1	1	0	0	1	1	1	0	0	8	57.1
Maas et al. [19]	1	1	1	1	1	1	1	0	1	1	1	1	1	12	85.7
Mitchell et al. [37]	1	1	1	2	1	1	1	1	1	1	1	1	1	14	100.0
Mo and Chow [14]	1	1	1	2	1	1	1	0	1	1	1	0	0	11	78.6
Mo and Chow [20]	1	1	0	1	1	1	1	0	1	1	1	0	0	9	64.3
Mo et al. [38]	1	1	1	2	0	1	1	1	1	0	1	0	0	10	71.4
Möhler et al. [22]	1	1	0	1	1	1	1	1	1	1	1	0	0	10	71.4
Nakayama et al. [42]	1	1	1	2	1	1	0	1	1	1	1	1	0	12	85.7
Quan et al. [15]	1	1	1	2	0	1	1	1	1	1	0	0	0	10	71.4
Quan et al. [43]	1	1	1	2	1	1	1	1	1	1	1	1	0	13	92.9
Schmitz et al. [13]	1	1	1	2	1	1	1	1	1	1	1	0	1	13	92.9
Seminati et al. [44]	1	1	1	2	1	1	0	0	1	1	1	0	0	10	71.4
Strohrmann et al. [34]	1	0	0	0	0	0	1	0	1	0	0	1	0	4	28.6
Zagatto et al. [46]	1	1	1	1	1	1	1	0	1	1	1	0	1	11	78.6

Questions: 1. Is the hypothesis/aim/objective of the study clearly described? 2. Are the main outcomes to be measured clearly described in the Introduction or Methods section? 3. Are the characteristics of the patients included in the study clearly described? 5. Are the distributions of principal confounders in each group of subjects to be compared clearly described? 6. Are the main findings of the study clearly described? 7. Does the study provide estimates of the random variability in the data for the main outcomes? 10. Have actual probability values been reported (e.g. 0.035 rather than < 0.05) for the main outcomes? 11. Were the subjects asked to participate in the study representative of the entire population from which they were recruited? 16. If any of the results of the study were based on “data dredging”, was this made clear? 18. Were the statistical tests used to assess the main outcomes appropriate? 20. Were the main outcome measures used accurate (valid and reliable)? 25. Was there adequate adjustment for confounding in the analyses from which the main findings were drawn? 27. Did the study have sufficient power to detect a clinically important effect where the probability value for a difference being due to chance is less than 5%?

% = percentage of total possible points

### Data Extraction

For each included study, one reviewer (RR) independently extracted information pertaining to runner group classification, the experience-related research question, measured biomechanical variables, task performed, and findings related to running experience, which were confirmed by a second reviewer (GAD). Only data related to conditions and comparisons described in the inclusion criteria were extracted (e.g., if running was performed in both traditional and minimalist footwear, only the data relating to the traditional footwear condition were included). Units for running distance and speed were reported in kilometers (km) and meters per second (m/s), respectively, and were converted when necessary. Studies were organized and presented in Table 3 according to the type of primary dependent variable. Kinematics, kinetics, spatiotemporal measurements were also organized by traditional (e.g., joint angles, ground reaction force (GRF), step length, etc.) and systems-based analysis (e.g., Lyapunov exponent, continuous relative phase, vector coding), which were organized into the following categories according to the authors’ description of the analysis: fluctuations/complexity, coordination, coordination variability, and variability/stability (Table 3).

**Table 3:** Experience group classification criteria, main research question related to running experience, task performed, variables evaluated, and main findings of each study included in the review

Study	Group Classification	Experience-related research question	Task	Variables	Main findings related to experience
**Traditional approach**					
*Kinematics, kinetics and spatiotemporal*					
Hreljac [36]	*Runners:* Middle to long distance runners who participated in running events on a competitive basis (n: 6 M, 6F) *Non-runners:* Participants that take part in sports but do not run (n: 6 M, 6F)	Does smoothness, as measured by jerk-cost, change between runners and non-runners?	Treadmill running at 3.35 m/s	*Traditional kinematics* Components of heel jerk (horizontal, vertical, and resultant) in the sagittal plane (2D) *Instrument:* 2D Video camera	*Traditional kinematics* During swing and entire stride, all components of jerk were lower for the runner group. During stance, only the vertical component of jerk was lower for runners.
Strohrmann et al. [34]	*Expert: *Over 45 km/week (n: 3) *Advanced:* Between 25 and 45 km/week (n: 6) *Intermediate:* Between 5 and 25 km/week (n: 6) *Beginner:* Between 0 and 5 km/week (n: 6) Sex: Not reported	Do changes in running biomechanics with fatigue (in treadmill or overground running) depend on a runners’ skill level?	Treadmill and overground running at a speed equivalent to 85% of the final speed in an incremental maximum test	*Traditional kinematics* Vertical oscillation, arm movement, trunk forward lean, heel lift, shoulder rotation, maximum knee flexion velocity, foot strike type and impact acceleration on upper body (shock attenuation) *Traditional spatiotemporal* Step frequency, contact time (normalized by step time) *Instrument:* Accelerometer	*Traditional kinematics* With fatigue, beginners and intermediate runners had more vertical oscillation, and higher impact acceleration on the upper body (shock attenuation). *Traditional spatiotemporal* With fatigue, beginners and intermediate runners had longer foot contact time.
Seminati et al. [44]	*Skilled:* Trained at least 6 h/week (over 3 times/week) and participated in marathons (n: 5 M) *Occasional:* Trained between 2 and 6 h/week (over 3 times/week) and had participated in half-marathons or 10 k (n: 7 M) *Untrained:* Practiced sports less than 2 h/week (3 times/week) (n: 7 M)	Are asymmetries in running different between skilled, occasional, and untrained runners?	Treadmill running at six different speeds from 2.22 to 5 m/s	*Traditional kinematics* Body’s Center of Mass global symmetry index *Instrument:* Motion capture	*Traditional kinematics* Untrained runners had lower body’s center of mass symmetry that the other groups at all speeds.
Boyer et al. [26]	*Higher mileage:* Over 32.2 km/week for at least one year (n: 13 M, 12F) *Lower mileage:* Less than 24.2 km/week with no history of higher mileage running (n: 13 M, 12F)	Are there differences in the Principal Components (PCs) weighting coefficients between higher and low mileage runners which are indicative of differences in the frontal and transverse plane joint kinematic waveform patterns and altered multisegment coordination?	Overground running at 3.5 m/s	Principal component analysis using: *Traditional kinematics* 3D motion of pelvis, hip, knee, ankle, mid-to-rearfoot, fore-to-midfoot and hallux-to-forefoot segments *Traditional kinetics* Three GRF components and two COP components *Instrument:* Motion capture, Force plate	Differences between groups in four PCs, which explained 4.6% of the data variation. *Traditional kinematics* In the transverse plane: Lower mileage runners had more externally rotated hip through terminal stance, less pelvic transverse plane rotation throughout ground contact and greater rearfoot external rotation. In the frontal plane: Lower mileage group had more knee adduction and less hip adduction throughout the stance and smaller rearfoot inversion angle in the final two-thirds of stance. In the sagittal plane: Lower mileage group showed less forefoot to midfoot flexion throughout stance. *Traditional kinetics* Ground reaction force and center of pressure components were not part of the linear combination of PCs that differed between groups.
Schmitz et al. [13]	*Experienced:* At least 19 km/week for the last year (n: 19F) *Novice:* No running for the past 5 years (n: 19F)	Are there differences in running mechanics between experienced and novice runners?	Treadmill running at 3.3 m/s	*Traditional kinematics* Peak frontal and transverse hip angles *Traditional kinetics* Vertical impact peak and loading rate *Instrument:* Motion capture, Force plate	*Traditional kinematics* No significant differences were found between groups for all kinematic variables. However, effect size analysis indicates that novice runners tended toward more hip internal rotation. *Traditional kinetics* No significant differences were found between groups for all kinetic variables.
Boey et al. [16]	*Well-trained runners:* Ran more than 50 km/week for at least two years under coach supervision (n: 6 M, 5F) *Recreational runners:* Ran between 10 and 30 km/week for at least six months (n: 6 M, 6F) *Untrained participants:* No running experience and did less than two hours of sports per week (n: 6 M, 6F)	Is vertical acceleration of the tibia influenced by running surface, running speed, and running experience?	Running overground at self-selected or fixed speed on concrete, synthetic running track or woodchip trail	*Traditional kinematics* Positive vertical acceleration peak *Instrument:* Accelerometer	*Traditional kinematics* No differences in vertical acceleration peak between the three groups when running at either speed in any surface.
Agresta et al. [3]	*No groups*: distance runners with experience as a continuous variable based on years (n: 50 M, 50F)	Are running biomechanics related to injury associated with years of running experience?	Treadmill running at self-selected speed	*Traditional kinematics* Peak knee flexion, knee adduction, hip internal rotation and trunk flexion during contact, foot strike angle and knee flexion at foot strike *Traditional kinetics* Vertical impact peak, loading rate and active peak *Traditional spatiotemporal* Stride rates, stride time, stride length, contact time and flight time *Instrument*: Inertial Measurement Unit, Pressure plate	*Traditional kinematics* Running experience was not significantly associated with kinematic variables. *Traditional kinetics* Running experience was not significantly associated with kinetic variables. *Traditional spatiotemporal* Running experience was not significantly associated with spatiotemporal variables.
Maas et al. [19]	*Competitive* At least 3 years of running experience, average weekly distance of 70 km or 50 km (for males and females, respectively) and participation in running competitions (n: 10 M, 5F) *Novice*: Weekly distance under 10 km and no history of competitive running (n: 9 M, 6F)	Do competitive and novice runners alter their kinematics differently with fatigue?	Treadmill running at a 3.2 km trial pace until exhaustion	*Traditional kinematics* Peak values and whole time-series of ankle, knee, hip, pelvis, and trunk kinematics in the three planes *Instrument:* Motion capture	*Traditional kinematics* With exhaustion, peak trunk flexion increased more for the novice group. With exhaustion, the time-series curves of novices shifted towards more hip abduction early in the swing phase, while the opposite was true for competitive runners.
Harrison et al. [18]	*Recreational:* At least 16 km/week for at least 12 months (n: 10F) *Novice:* No regular running in the past 12 months (less than 16 total km) (n: 10F)	Does the frontal and transverse plane time series kinematic differ between novice and recreational runners?	Treadmill running at 2.7 m/s	*Traditional kinematics* Time-series of hip, knee and ankle angles in the frontal and transverse planes Relative change in angular position between hip and ankle using angle-angle plots *Instrument:* Motion capture	*Traditional kinematics* Experienced runners had greater eversion and tibial internal rotation throughout stance. Novice runners had greater knee abduction and internal rotation throughout stance and displayed less knee range of motion. Novice runners landed with more hip abduction and reached peak adduction earlier in stance. Novices were also more adducted by terminal stance. Experienced runners had more hip internal rotation throughout stance. Visual differences in the hip-ankle angle-angle plots were reported between groups.
Quan et al. [15]	*Experienced:* At least 5 years and 32 km/week (n: 12 M) *Novice:* Between 3.2 and 8 km/week with no formal experience or participation in running competition (n: 12 M)	Are there biomechanical differences between experienced and novice runners?	Overground running at 3.3 m/s	*Traditional kinematics* Sagittal hip, knee and ankle joint angles peaks, ranges of motion and peak angular velocity *Traditional kinetics* Sagittal hip, knee and ankle joint maximum and minimum internal moments and powers Average vertical loading rate and vertical instantaneous loading rate *Traditional spatiotemporal* Contact time *Instrument:* Motion capture, Force plate	*Traditional kinematics* There are inconsistencies in the reporting of the results between the figure, text and table. *Traditional kinetics* Ankle plantarflexion peak moment was higher for novices. Hip extension peak moment was lower for novices. Hip peak extension power was lower for novices. Vertical instantaneous loading rate was lower for novices. *Traditional spatiotemporal* No differences between groups for contact time.
Quan et al. [43]	*Competitive:* At least 5 years and 15 km/week (n: 20 M) *Recreational*: Between 3 and 5 km/week with no formal long-distance training (n: 20 M)	What are the differences in kinematics between competitive and recreational runners, using Principal Component Analysis?	Overground running at 3.3 m/s	*Traditional kinematics* Principal components obtained from ankle, knee, and hip kinematics in the three planes *Instrument:* Motion capture	*Traditional kinematics* Recreational runners had greater dorsiflexion than competitive runners at initial contact, but smaller during overall stance. Recreational runners also had smaller ankle inversion and greater frontal and transverse plane range of motion. Recreational runners had larger knee flexion, abduction and internal rotation angle and smaller transverse plane range of motion. Recreational runners had greater hip flexion, adduction and external rotation angles and smaller frontal plane range of motion.
Carter et al. [39]	*Experienced:* 25 km/week with a 10 km time of < 40 min (n: 10 M) *Novice***:** Recreationally active but no prior running engagement (n:10 M)	What are the biomechanical differences between experienced and novice runners that enable their successful group classification using a support vector machine algorithm?	Treadmill running at 10 km/hr	*Traditional kinematics* Upper arm and T10 linear acceleration and angular velocity time-series *Instrument:* Inertial measurement units embedded with tri-axial accelerometer and gyroscope	*Traditional Kinematics* Experienced runners had greater upper arm rotation velocity along the anterior–posterior axis between 2–17%, 25–32%, 49–58%, and 69–87% of the stride cycle, and more anterior–posterior trunk deceleration during mid-stance than novice runners.
Fadillioglu et al. [23]	*Experts:* At least 50 km/week in the previous eight weeks, membership of a running club for at least 2 years and a 10 k best time under 35 min (n: 13 M) *Novices:* No more than one running session per week and never have trained in a running club or for a running event (n: 12 M)	How do biomechanical parameters and their variability changes between runners with difference expertise levels?	Treadmill running at 2.8 or 4.2 m/s	*Traditional kinematics* Vertical oscillation of center of mass *Traditional spatiotemporal* Stride frequency and duty factor (step time relative to the flight time) *Variability* Vertical oscillation of center of mass, stride frequency and duty factor coefficient of variation	*Traditional kinematics* Vertical oscillation of center of mass was not different between groups. *Traditional spatiotemporal* Novices had a higher duty factor than experienced runners. There were no differences between groups for stride frequency. *Variability* Variability of vertical oscillation of center of mass and stride frequency was higher for NOV than for EXP.
Jiang et al. [45]	*Experienced:* Over 3 years and at least 30 km/week (n: 15 M) *Novices:* Between 2–10 km/week and not take part in training or competition (n: 15 M)	What are the differences between groups in joint motion during a 5 km run?	Overground running at self-selected speed	*Traditional kinematics* Principal component analysis using 3D motion of hip, knee and ankle Discrete values of joint range of motion *Traditional kinetics* Principal component analysis using 3D moments of hip, knee and ankle and 3 GRF axes Discrete values of peak joint moment, peak propulsive GRF, peak braking GRF, impact peak of vertical GRF and vertical average loading rate of GRF *Instrument:* Motion capture, Force plate	*Traditional kinematics* Differences between groups using principal component analysis of joint angles were found for ankle in the sagittal and frontal plane, knee in all planes and hip in all planes. In the discrete analysis, experienced group had greater range of motion for knee extension/flexion, and smaller range of motion for ankle inversion/eversion and hip adduction/abduction. *Traditional kinetics* Differences between groups using principal component analysis were found in joint moments for ankle in the three planes, knee in the three planes, hip in the frontal and transverse planes and for GRF in the antero-posterior and medial–lateral axis. In the discrete analysis, experienced group had greater peak joint moment of hip extension and abduction and smaller peak joint moment of ankle inversion and internal rotation. Experienced group also had greater impact peak of vertical GRF and smaller peak propulsive GRF.
*Traditional kinetics and spatiotemporal*					
Lees and Bouracier [41]	*Runners:* Between 40 and 110 km/week (n: 7 M) *Non-runners:* Played sports (n: 7 M)	Do ground reaction force characteristics change between runners and non-runners?	Overground running at preferred speed	*Traditional kinetics* Vertical impact force peak, vertical force slope (loading rate), negative impulse and positive impulse *Instrument:* Force plate	*Traditional kinetics* Negative and positive impulses were smaller for runners.
Mo et al. [38]	*Competitive:* World Masters Association Age Grade higher than 60% (n: 7 M, 4F) *Recreational:* World Masters Association Age Grade lower than 60% (n: 5 M, 4F) *Novice:* Regular running practice for less than two years of at ≥ 20 km/week, 3 times per week with minimum of 30 min per time OR runners that never participated in any race (n: 6 M, 5F)	Is bilateral asymmetry different between runners of different levels at different running speeds?	Treadmill running at five speeds between 2.2 and 3.3 m/s	Bilateral asymmetry (Symmetry index) of the following variables: *Traditional kinetics* Peak vertical ground reaction force, peak braking and propulsion forces, time to the peaks, vertical average loading rate and vertical instantaneous loading rate *Traditional spatiotemporal* Stride, step, stance, and flight times *Instrument:* Instrumented treadmill	*Traditional kinetic* There were no differences between groups for symmetry index of the kinetic variables. There was an interaction between speed and experience for vertical average loading rate and time to peak vertical ground reaction force, where the symmetry index varied differently between groups as speed increased. *Traditional spatiotemporal* There were no differences between groups for symmetry index of the spatiotemporal variables.
Mitchell et al. [37]	*Collegiate:* Running in the previous year for an intercollegiate team (n: 24 M, 6F) *Recreational:* At least 16 km/week but running up to 3 times a week (n: 24 M, 6F) *Control:* Participants that do not run but were physically active for at least 30 min, 3 times per week (n: 24 M, 6F)	How do knee frontal and sagittal moments differ between recreational runner and a control group?	Overground running at self-selected running speed	*Traditional kinetics* Peak external knee flexion and knee adduction moment *Instrument*: Motion capture, Force plate	*Traditional kinetics* No differences between the control group and recreational runners. Collegiate runners had greater peak knee flexion moments and lower peak adduction moments than the control group.
Zagatto et al. [46]	*Runners:* Training for at least 2 years (n: 18 M) *Physically active:* Untrained but performing sporadic physical activities (17 M)	How do mechanical variables differ between amateur runners and physically active participants during a supramaximal exhaustive run?	Treadmill running at 115% of the velocity associated with VO_2max_	*Traditional kinetics* Total work, external work, internal work, vertical work, horizontal work, trunk internal work, upper internal work, lower internal work, total power, external power, internal power, based on the center of mass trajectory *Traditional spatiotemporal* Stride frequency, stride length, flight time, contact time *Instrument*: Motion capture	*Traditional kinetics* Runners had lower vertical work than physically active participants. *Traditional spatiotemporal* Runners had longer stride length and shorter contact phase duration.
*Traditional spatiotemporal*					
Gómez-Molina et al. [33]	*Trained: *At least two years of running training, 3 weekly sessions and best half marathon time between 70 and 86 min (n: 10) *Untrained:* No specific running training but physically active (n: 11) Sex: Not reported	How do spatiotemporal parameters change between trained and untrained participants?	Treadmill running at three set speeds (trained: 3.1, 3.6, 4.2 m/s; untrained: 2.5, 3.1, 3.6 m/s) And treadmill running in a graded exercise test (starting at 1.7 m/s)	*Traditional spatiotemporal* Step rate, step length, contact time and flight time *Instrument:* Contact laser platform	*Traditional spatiotemporal* Trained group showed higher step rate and shorter step length than the untrained group. No difference for contact and flight time.

**Systems-based analyses**					
*Fluctuation, stability and variability*					
Nakayama et al. [42]	*Runners:* Member of track and field running teams (n: 7 M) *Non-runners:* No specific running training (n: 6 M) Sex: All males	Are there differences in gait cycle variability and fluctuations between trained runners and untrained non-runners?	Treadmill running at 80, 100 and 120% of self-selected speed	*Fluctuation* Long-range correlations of stride interval fluctuations, measured with detrended fluctuation analysis (α) *Variability* Stride interval coefficient of variation *Traditional spatiotemporal* Stride interval *Instrument:* Footswitches	*Fluctuation* Long-range correlations were bigger for non-runners. *Variability* Stride interval variability was higher for non-runners than runners. *Traditional spatiotemporal* No differences in stride interval between groups (after removing the effects of speed).
Mo and Chow [14]	*Experienced***:** At least four years and 30 km/week of running (n: 14 M, 3F) *Novice:* Less than six months of running training and distance below 20 km/week (n: 15 M, 2F)	Are stride-to-stride variability and complexity different between experienced and novice runners during a prolonged run?	Treadmill running at anaerobic threshold speed	*Fluctuation (Complexity)* Long-range correlations of stride interval measured with Detrended fluctuation analysis (α) *Variability* Coefficient of variation for stride interval *Traditional spatiotemporal* Stride interval *Instrument:* Motion capture	*Fluctuation (Complexity)* Long-range correlations behaved differently between groups at certain parts of the run, but there were no overall differences between them. *Variability* Experienced runners varied the coefficient of variation throughout the run, while novices did not. *Traditional spatiotemporal* No difference between groups for mean stride interval.
Agresta et al. [21]	*Experienced:* 10 years or more (n: 17) *Intermediate:* between 4 and 9 years (n: 11) *Novice:* 3 years or less (n: 10) Sex: Not reported	How are stride-to-stride fluctuations and adaptive response to perturbations different between runners with different experience levels?	Treadmill running at self-selected speed while matching metronome beats and then in silence	*Fluctuation* Long-range correlations for step rate and contact time, measured with Detrended fluctuation analysis (α) *Instrument:* Instrumented treadmill	*Fluctuation* Experienced runners had lower step rate and contact time long-range correlations at baseline. Experienced runners were most successful at returning to their preferred step frequency after perturbation.
Frank et al. [40]	*Trained:* At least 30 km/week (n: 12 M) *Novice:* Less than a total of 10 km within the past year (n: 12 M)	Is joint level local dynamic stability difference between trained and novice runners?	Treadmill running at self-selected speed	*Stability* Ankle, knee, and hip variability in the sagittal plane, measured with local dynamic stability using the largest Lyapunov exponent *Instrument:* Motion capture	*Stability* Novices had less ankle, knee, and hip sagittal stability than trained runners.
Brahms et al. [17]	*Elite Group:* At least 4 weekly sessions for the past two years and competed at provincial or intercollegiate level (n: 16) *Recreational:* Running participation for up to 3 h/week but no specific competitive running training (n: 16) Sex: Not reported	Are the effects of fatigue on stride pattern variability and Long-range correlations different between experience levels?	Overground running at a speed of subjects’ 5 k time	*Fluctuation* Long-range correlations of stride time, stride length, contact time and peak impact acceleration in the beginning, middle and end of a fatiguing run, measured with Detrended fluctuation analysis (α) *Variability* Coefficient of variation of the same variables *Traditional kinematics/spatiotemporal* Mean values of the same variables *Instrument:* Inertial Measurement Unit	*Fluctuation* Long-range correlations did not differ between groups. *Variability* Recreational runners had greater stride time variation than elite. *Traditional kinematics/spatiotemporal* Recreational runners displayed smaller stride length, contact time and peak impact acceleration, all of which are speed dependent.
*Coordination and coordination variability*					
Floría et al. [47]	*Runners***:** At least 5 weekly sessions in the previous 12 months (n: 10F) *Non-runner***:** No running training and no more than 2 days a week of recreational running (n: 12F)	Is there a difference in joint coordinative variability (using continuous relative phase) in gait between trained runners and non-runners?	Treadmill running at self-selected speed	*Coordination* In-phase and out-of-phase coupling of HIP_sagittal_-KNEE_sagittal_, HIP_frontal_-KNEE_sagittal_, KNEE_sagittal_-ANKLE_sagittal_ and KNEE_sagittal_-ANKLE_frontal,_ using continuous relative phase *Coordination variability* Variability of the coordination metrics *Instrument:* Motion capture	*Coordination* No significant differences between groups for the continuous relative phase values. *Coordination variability* No significand differences between groups in variability of the coupling pairs analyzed.
Hafer et al. [35]	*More-experienced:* Running consistently for more than 10 years (n: 16 M, 4F) *Less-experienced:* Running consistently for no more than 2 years (n: 17 M, 4F)	Does segment coordination and coordination variability differ between less-experienced and more-experienced runners?	Treadmill running at self-selected speed	*Coordination* Sagittal plane Thigh x Shank and Shank x Foot couples, using vector coding *Coordination variability* Variability of the coordination metrics *Instrument:* Inertial Measurement Unit	*Coordination* No differences in coordination between groups. *Coordination variability* Less-experienced runners had lower coordination variability for both couples (Thigh x Shank and Shank x Foot) during early and mid-stance.
Mo and Chow [20]	*Experienced:* At least four years and 30 km/week of running (n: 17) *Novice:* Less than six months of running training (n: 17) Sex: Not reported	Are the characteristics of lower-limb coordination and coordination variability different between experienced runners and novices during a prolonged run?	Treadmill running at anaerobic threshold speed	*Coordination* Coordination of the sagittal hip-knee and knee-ankle joint angle couples, and the sagittal pelvis-thigh, thigh-shank and shank-foot segment angle couples, using a vector coding approach *Coordination variability* Variability of the coordination metrics *Traditional kinematics* Peak sagittal angle, angle at initial contact and toe-off, range of motion and time to peak angle for ankle, knee and hip joints and foot, shank, thigh, and pelvis segments *Instrument:* Motion capture	*Coordination* Hip-knee anti-phase motion during midstance and pelvis-thigh in-phase motion during midstance and during total stance changed differently between groups. *Coordination variability* Experienced runners presented more coordination variability for hip-knee during terminal stance and for shank-foot during stance phase then novices. *Traditional kinematics* Time to peak hip sagittal angle decreased for the experienced group during the run but remained constant for novices.
Möhler et al. [22]	*Experts*: At least 50 km/week in the previous eight weeks, membership of a running club for at least 2 years and a 10 k best time under 35 min (n: 13) *Novice:* No more than one running session per week and never have trained in a running club or for a running event (n: 12) Sex: Not reported	Is movement variability structured differently in experienced athletes compared to novices?	Treadmill running at 2.8 or 4.2 m/s	*Coordination variability* Uncontrolled manifold analysis, using joint angles as the elemental variables which may change (UCM_⊥_) or may not change (UCM_||_) the performance variable (whole body Center of Mass trajectory) and the ratio between them (UCM_ratio_) *Instrument:* Motion capture	*Coordination variability* There were no UCM differences between groups in the 2.8 m/s speed At 4.2 m/s both UCM_||_ and UCM_⊥_ were higher for novices compared to experts, indicating that there was a higher variability but not a higher degree of stabilization of the center of mass trajectory.

Abbreviations: n is the sample size, M is males, F is females, m/s is meters per second, 2D is two-dimensional, km is kilometers.

Given the potential differences in running experience definitions across studies, the running experience groups from each study were organized into three tiers based on the connotation of the terminology used by the authors (e.g., upper tier: experienced/expert; medium tier: recreational/intermediate; lower tier: novice/beginner). Within each tier, the number of years and weekly distance cut-offs used in each study were ordered from high to low. The goal of this organization was to identify differences and overlap in the classification criteria between studies using similar classification methods (i.e., years or distance) (Table 4). Finally, to show the different interpretations of “participants who are not runners”, studies reporting that the less-experienced group did not engage in regular running were grouped and ordered from the most to least amount of running allowed, according to their inclusion criteria. For example, a study with the criterion “no more than 2-days a week of recreational running” included participants with more running allowed than a study with the criterion “no running for the past five years” (Table 5).

**Table 4:** Studies that used years of running experience and/or kilometers per week as the criteria for grouping runners by experience. Studies are grouped into tiers according to the connotation of the categorical terminology used to describe the groupings by the authors of each study. Within each category, cut-offs from each study are ranked from greatest to least based on years and kilometers per week

Years of experience								
Upper tier			Medium tier			Lower tier		
Study	Classification	Years	Study	Classification	Years	Study	Classification	Years
Agresta et al. [21]	Experienced	> 10	Agresta et al. [21]	Intermediate	4–9	Agresta et al. [21]	Novice	< 3
Hafer et al. [35]	More-experienced	> 10	Goméz-Molina et al. [33]	Trained	> 2	Mo et al. [38]	Novice	< 2
Quan et al. [15]	Experienced	> 5	Zagatto et al. [46]	Runner	> 2	Mo and Chow [20]	Novice	< 0.5
Quan et al. [43]	Experienced	> 5	Hafer et al. [35]	Less-experienced	< 2	Mo and Chow [14]	Novice	< 0.5
Mo and Chow [14]	Experienced	> 4	Boey et al. [16]	Recreational	> 0.5			
Mo and Chow [20]	Experienced	> 4						
Maas et al. [19]	Competitive	> 3						
Boey et al. [16]	Well-trained	> 2						
Brahms et al. [17]	Elite	> 2						
Mohler et al. [22]	Experts	> 2						
Fadillioglu et al. [23]	Experts	> 2						

Kilometers per week (km/w)								
Upper tier			Medium tier			Lower tier		
Study	Classification	km/w	Study	Classification	km/w	Study	Classification	km/w
Boey et al. [16]	Well-trained	> 50	Frank et al. [40]	Trained	> 30	Mo and Chow [14]	Novice	< 20
Maas et al. [19]	Competitive	> 50	Boyer et al. [26]	Lower Mileage	< 24	Maas et al. [19]	Novice	< 10
Mohler et al. [22]	Experts	> 50	Harrison et al. [18]	Recreational	> 16	Jiang et al. [45]	Novice	2–10
Strohrmann et al. [34]	Expert	> 45	Mitchell et al. [37]	Recreational	> 16	Quan et al. [15]	Novice	3–8
Lees and Bouracier [41]	Runners	40–110	Strohrmann et al. [34]	Intermediate	5–25	Strohrmann et al. [34]	Beginner	0–5
Boyer et al. [26]	Higher Mileage	> 32	Quan et al., [43]	Recreational	3–5			
Quan et al. [15]	Experienced	> 32						
Strohrmann et al. [34]	Advanced	25–45						
Mo and Chow [14]	Experienced	> 30						
Mo and Chow [20]	Experienced	> 30						
Jiang et al. [45]	Experienced	> 30						
Carter et al. [39]	Experienced	> 25						
Schmitz et al. [13]	Experienced	> 19						
Quan et al. [43]	Competitive	> 15

**Table 5:** Criteria used by studies that had the less-experienced group with no “formal” running training. Studies are listed according to the likelihood that the participants ran occasionally

Study	Criteria	Classification
Floría et al. [47]	No running training and *no more than 2 days a week of recreational running*	Non-runner
Möhler et al. [22]	No more than *one running session per week* and no formal training	Novices
Fadillioglu et al. [23]	No more than *one running session per week* and no formal training	Novices
Harrison et al. [18]	No regular running in the past 12 months *(less than 16 total km)*	Novice
Frank et al. [40]	Less than a *total of 10 km* within the past year	Novice
Seminati et al. [44]	Practiced *sports less than 2 h per week*	Untrained
Boey et al. [16]	No running experience and *less than two hours of sport per week*	Untrained
Mitchell et al. [37]	Do not run but *physically active for at least 30 min, 3 times per week*	Control
Gómez-Molina et al. [33]	No specific running training but *physically active for 2–3 days per week*	Untrained
Hreljac [36]	No running but *sport participation*	Non-runner
Lees and Bouracier [41]	*Played sports*	Non-runner
Nakayama et al. [42]	*No specific running training*	Non-runner
Carter et al. [39]	*No prior involvement* in distance running training	Novice
Schmitz et al. [13]	No running for the *past five years,* but physically active	Novice

## Results

**Fig. 1:**
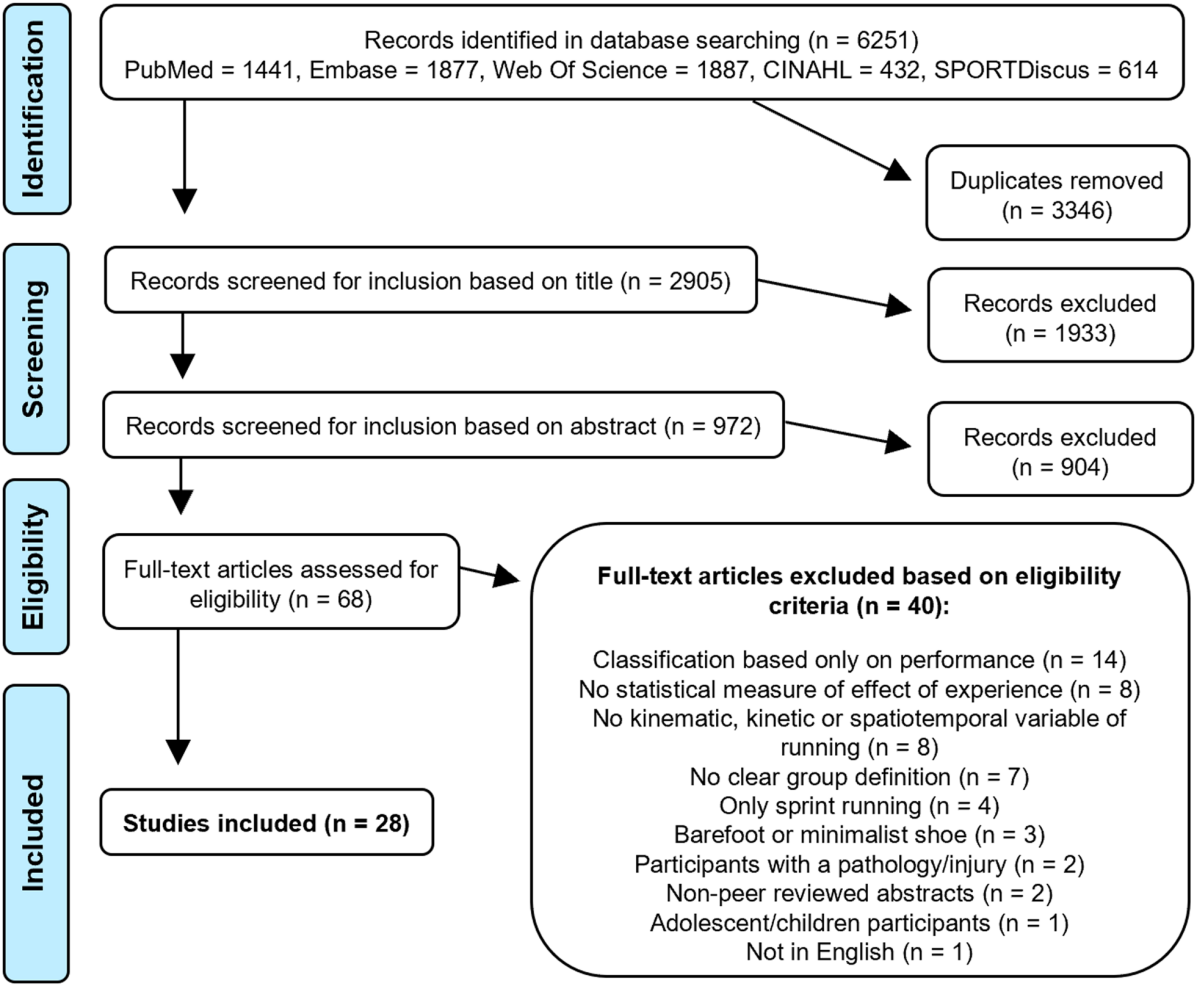
Flow diagram of the study selection procedure. Some articles were excluded based on more than one criterion in the full-text screening stage for eligibility

The initial search identified 6251 records of which 3346 were duplicates, and so a total of 2905 records were screened (Fig. 1). Title screening resulted in 972 studies that were selected for abstract screening. Sixty-eight studies were selected for full-text eligibility evaluation of which 40 were excluded. The main reasons for exclusion during full-text evaluation were the following: lack of statistical assessment for the effect of experience (n = 8); not evaluating a kinematic, kinetic, or spatiotemporal variable during running (n = 8); no clear experience group definition (n = 7); or including only a performance-based experience definition (n = 14). Therefore, a total of 28 studies examining 916 total subjects were included in this systematic review (Fig. 1). Using a modified version of the Downs and Black checklist, 22 studies were considered high quality and six were considered low quality; lack of power calculation and adjustment for confounders were the most common limitations (Table 2).

### Participants and Study Design

From the included 28 studies, a total of 930 participants were evaluated, with 521 males, 238 females, and six studies not reporting the sex [17, 20–22, 33, 34]. Nine studies evaluated both sexes [3, 14, 16, 19, 26, 35–38], ten studies evaluated only males [15, 23, 39–46], and 3 studies evaluated only females [13, 18, 47].

Terminology for the experience groups varied between studies. The terms used most were experienced (n = 7) [13, 15, 20, 21, 39, 45], novice (n = 13) [13–15, 18–23, 38, 40, 45, 47], recreational (n = 6) [16–18, 37, 38, 43], runners (n = 4) [36, 41, 42, 47], and non-runner (n = 4) [36, 39, 41, 42]. Terms used in three studies or less included: competitive [19, 38, 43], expert [22, 23, 34], intermediate [21, 34], trained [33, 40], and untrained [16, 33, 44].

The mode of running to measure gait included treadmill (n = 19) [3, 13, 14, 18–23, 33, 35, 36, 38–40, 42, 44, 46, 47], overground (n = 8) [15–17, 26, 37, 41, 43, 45], and both (n = 1) [34]. The testing speeds were self-selected (n = 9) [3, 16, 21, 35, 40–42, 45, 47], standardized (n = 14) [13, 15, 16, 18, 22, 23, 26, 33, 36–39, 43, 44], and equal to a performance metric (n = 6; e.g., best 5 km speed, anaerobic threshold, etc.) [14, 17, 19, 20, 34, 46]. Median standardized speed was 3.3 m/s (range = 2.7–3.5 m/s), median self-selected speed was 3.2 m/s (range = 2.7–4.95 m/s), and median speed based on a performance metric was 3.8 m/s (range = 3.1–5.1 m/s).

The instrumentation used to measure the variables of interest were three-dimensional motion capture (n = 16) [13–15, 18–20, 22, 23, 26, 37, 40, 43–47], inertial measurement units or accelerometers (n = 6) [3, 16, 17, 34, 35, 39], in-ground force platforms (n = 6) [13, 15, 26, 37, 41, 45], and instrumented treadmill (n = 2) [21, 38]. The following were each used in one study: footswitches [42], contact laser platforms [33], pressure plates [3], and two-dimensional video camera.

Most studies assessed multiple variables using traditional and/or alternative analysis approaches. Reducing time-series data to one single value variable (i.e., peak, mean, range of motion, maximum/minimum values, time to peak) was done in 13 studies. Time-series analysis (statistical parametric mapping, principal component analysis, cubic splines) was used in 6 studies. Systems-based analyses were adopted in 9 studies (fluctuation/complexity = 4 [14, 17, 21, 42], variability/stability = 5 [14, 17, 23, 40, 42], coordination = 3, coordination variability = 4 [20, 22, 35, 47]). Systems-based analyses included long-range correlations, largest Lyapunov exponent, continuous relative phase, vector coding and uncontrolled manifold analysis.

A meta-analysis was not possible because no more than 5 studies measured the same variable [3, 15, 17, 33, 38], but often without the same data collection and analysis methods. Therefore, the following sections present the main findings organized by variable type. Due to the variability in group classification, the terms more-experienced and less-experienced were used when reporting study results instead of the terminology used in each study.

### Traditional Kinematics

Of the 16 studies that evaluated kinematics (Table 3) [3, 13, 15–20, 23, 26, 34, 36, 39, 43–45], 11 studies used a single value to represent the kinematic variable (e.g., peak joint angles, ranges of motion) and performed common statistical analyses (e.g., t-tests, analysis of variance, linear regression) [3, 13, 15, 16, 19, 20, 23, 34, 36, 44, 45], while 6 studies evaluated the whole time-series using principal component analysis, cubic splines, and statistical parametric mapping for statistical analyses [18, 19, 26, 39, 43, 45].

Using discrete variables and traditional analysis methods, 4 studies found no statistical effect of experience on kinematics [3, 13, 16, 23] whereas 6 studies found an experience effect for the following variables [17, 19, 20, 34, 36, 44]: compared with more-experienced runners, less-experienced runners ran with greater jerk at the heel [36], less body center of mass symmetry between right and left steps [44], lower peak impact acceleration at participants’ 5 km time [17], greater vertical oscillation and upper body impact acceleration with fatigue [34], and greater peak trunk flexion with fatigue [19]. In addition, time to peak sagittal hip angle was maintained with fatigue in less-experienced runners but decreased with fatigue in more-experienced runners [20]. Quan et al. [15] also reported significant differences between groups in peak sagittal joint angles, but the results are challenging to interpret due to inconsistencies between the text, tables, and figures.

The 6 studies that used a time-series analysis of kinematics found significant effects of experience, but evaluated different variables [13, 17, 22, 41, 45, 47] (Table 3). The methods included statistical parametrical mapping [19, 39, 46], cubic splines [18], and principal component analysis [26, 39, 43]. The significantly different comparisons are presented in Table 3.

### Kinetics

Traditional methods were used in 7 studies to assess ground reaction force and/or center of pressure variables (Table 3) [3, 13, 15, 26, 38, 41, 45]. Three studies found no effect of experience on the peak impact force, loading rate [3, 13], or peak active force within the vertical component of the ground reaction force [3], while 1 study found greater impact peak in the more-experienced runners [45]. Similarly, using a principal component analysis of the time-series, no significant differences between experience groups were observed for each component of the three-dimensional ground reaction force or the two components of center of pressure [26]. In addition, no significant differences were found for bilateral asymmetry of five ground reaction force metrics [38]. However, three studies found that less-experienced runners had significantly greater antero-posterior braking and propulsive impulses [41], greater peak propulsive ground reaction force [45] and lower vertical instantaneous loading rate [15] than more-experienced runners. Studies normalized GRF values by bodyweight or body mass [3, 13, 15, 45] or did not mention normalization [26, 38, 41].

Three studies evaluated joint kinetics [15, 37, 45]. Mitchell et al. [37] found smaller peak external knee flexion moments and larger peak external knee adduction moments (normalized by bodyweight x height) in less-experienced runners compared with more-experienced runners. Quan et al. [15] found that the less-experienced runners had less peak internal hip extension power, smaller hip extension moment, and greater internal ankle plantarflexion moment than the more-experienced runners (normalized by body mass). Peak ankle and knee power and peak knee moments were not significantly different between groups [15]. Additionally, one study used principal component analysis to find that less-experienced runners had larger ankle inversion, ankle internal rotation, and hip flexion moments (normalized by body mass) throughout stance, as well as greater hip external rotation in early stance [45].

One study compared several work and power variables normalized by bodyweight based on the center of mass trajectory, finding that more-experienced runners ran with less vertical work than the less-experienced group [46].

### Spatiotemporal

Ten studies measured spatiotemporal variables (Table 3). Running experience did not affect foot contact time in 4 studies [3, 15, 33, 38]. Conversely, less-experienced runners had a greater normalized foot contact time (percentage of step) after an exhausting run compared with more-experienced runners [34]. Although Brahms et al. [17] and Zagatto et al. [46] also observed an experience effect on foot contact time (shorter in Brahms et al. [17] and longer in Zagatto et al. [46] for less-experienced), running speed was not standardized which may have influenced the results.

Non-normalized step frequency [33, 34] and duty factor (step time relative to flight time) [23, 38] had conflicting results. Stride and step length also presented conflicting results in four studies, albeit using different speeds and normalization techniques [3, 17, 33, 46]. Other variables that were measured but showed no effect of experience were: stride frequency normalized by leg length [23], non-normalized stride frequency [3], step time [38], stride time/interval [3, 14, 17, 38, 42], and flight time [3, 33, 38].

### Fluctuation/Complexity and Variability

Four studies evaluated the effect of experience on the fluctuation of running pattern variables using detrended fluctuation analysis (Table 3) [14, 17, 21, 42]. Nakayama et al. [42] found that less-experienced runners had stronger long-range correlations (LRC) of stride interval when running at three different speeds than more-experienced runners. Agresta et al. [21] found that more-experienced runners had lower step rate and contact time LRCs than less-experienced runners during unperturbed running and were more successful at returning to their preferred step frequency after an enforced step frequency perturbation. Mo and Chow [14] measured stride interval LRCs during a prolonged run at anaerobic threshold speed finding that LRC behaved differently between groups during certain parts of the run, but there were no overall significant differences between groups. Similarly, Brahms et al. [17] evaluated LRCs of stride interval (i.e., stride time), contact time, stride length, and peak impact acceleration during a prolonged run, finding no effect of experience on any variable.

The variability of spatiotemporal and kinematic variables was evaluated in four studies [14, 17, 23, 42]. Less-experienced runners had greater stride interval variability [17, 42], vertical center of mass oscillation variability, and stride frequency variability [23] than more-experienced runners. Conversely, Mo and Chow [14] found that more-experienced runners varied their stride interval coefficient of variation throughout a prolonged run, while less-experienced runners did not.

One study investigated the effect of experience on local dynamic stability of lower limb sagittal joint angles using the largest Lyapunov exponent, finding less-experienced runners had lower stability than more-experienced runners [40].

### Coordination and Coordination Variability

Three studies compared coordination of lower limb joint or segment angles between running experience groups using either vector coding or continuous relative phase (Table 3) [20, 35, 47]. Floría et al. [47] and Hafer et al. [35] found no differences between experience groups for the coupling pairs analyzed using continuous relative phase and vector coding, respectively. However, also using vector coding, Mo and Chow [20] found that sagittal hip-knee anti-phase and pelvis-thigh in-phase motion during mid-stance and pelvis-thigh in-phase motion across the stance phase changed differently between groups throughout a prolonged run. All three studies also evaluated coordination variability. Hafer et al. [35] found lower sagittal plane thigh-shank and shank-foot coordination variability during early and mid-stance while Mo and Chow [20] found less-experienced runners had lower coordination variability for hip-knee coupling during terminal stance and shank-foot coupling during the stance phase than more-experienced runners. Floría et al. [47], however, found no influence of experience on coordination variability.

Möhler et al. [22], used an uncontrolled manifold analysis to evaluate how joint angles influenced the body center of mass variability, finding that less-experienced runners had greater variability but not a greater degree of stabilization of the center of mass trajectory than more-experienced runners.

### Classification Criteria

Five main criteria were identified to classify participants into running experience groups used in isolation or in combination: weekly distance (n = 18 [13–16, 18–20, 22, 23, 26, 34, 37–41, 43, 45]), years of running (n = 15 [3, 14–17, 19–23, 33, 35, 38, 43, 46]), participation in competitions (n = 8 [15, 17, 19, 36–38, 42, 44]), number of weekly running sessions or hours of running (n = 6 [17, 33, 37, 38, 44, 47]), and performance (n = 5 [22, 23, 33, 38, 39]; e.g., best race time). Fourteen studies reported that participants in the less-experienced group were not runners [13, 16, 18, 22, 23, 33, 36, 37, 39–42, 44, 47]. Table 4 lists the studies that used years of running experience and/or kilometers per week as the criteria for grouping runners by experience, as defined by the authors of each study. The studies are organized into tiers (upper, medium, and lower) based on the connotation of the terminology used for each experience group. The inclusion criteria for studies where participants in the less-experienced group were not runners are presented in Table 5.

## Discussion

One aim of this systematic review was to synthesize the current literature examining the effects of running experience on gait biomechanics given that experience-related differences in gait could explain the elevated risk of RRI in less-experienced runners compared with more experienced runners. The systematic review included 28 studies evaluating the effects of running experience on kinematics, kinetics, and spatiotemporal variables analyzed traditionally and/or using systems-based analyses. Most studies found statistically significant differences between groups for some variables; however, there were inconsistencies between studies evaluating similar variables regarding whether statistical significance was found. Several gait variables were measured in only one study which prevents conclusions regarding generalizability or consistent effects. This lack of consistency and replication makes it difficult to identify clear differences in gait biomechanics between more- and less-experienced runners, and makes it challenging to link gait differences with differing rates of RRI between experience levels.

The second aim was to evaluate the current criteria and terminology used to classify running experience. The definitions used to classify experience groups were inconsistent and highly variable across the included studies. The inconsistency in experience group classification likely explains the lack of consistent findings for similar gait variables. The variability in experience definitions and the lack of replication makes the existing literature too heterogeneous to complete a meta-analysis and draw a definitive conclusion regarding the effect of running experience on running mechanics. These issues indicate the need for a standardized classification procedure for running experience. A standardized taxonomy to classify running experience for future research is proposed later in this discussion.

### Quality of Studies and Factors Influencing Synthesis of Results

Most studies were considered to be high quality according to the Downs and Black checklist [24] because the aims, outcomes, measures of data variability, and statistical analysis were clearly stated. Despite being high quality, issues that may contribute to mixed results or the inability to compare between studies include small sample sizes, not reporting participant sex, different methodology (e.g., criterion speed versus preferred running speed), and reporting only the gait response to an intervention (e.g., change due to a fatiguing run) rather than the baseline value.

Five studies reported a priori sample size estimates and no studies reported power calculated post-hoc [13, 19, 37, 45, 46]. It is possible that the null findings in several studies were due to an insufficient sample size within each running experience group.

Six studies failed to report the participants’ sex [17, 20–22, 33, 34]. Sex is known to influence running biomechanics [5, 25, 27] such as hip adduction, hip internal rotation, and knee abduction [27]. Thus, not reporting the distribution of sex within each experience group or not accounting for sex-based differences could have contributed to mixed results between studies and makes the interpretation of experience-effects challenging.

The choice of standardized or preferred speeds adds to between-study and within-study variability. Given that running speed influences biomechanics [29, 48] and more experienced runners tend to run faster, some studies compared groups running at their preferred speed then statistically controlled for speed effects [3, 42]. Conversely, other studies incorporating different speeds between groups or participants did not statistically control for speed or did not report doing so [17, 34, 37, 46] and so the observed differences between experience groups may be an effect of speed rather than running experience. Standardizing running speed across participants minimizes speed effects, but incorporating preferred speeds ensures that runners are evaluated at familiar and comfortable speeds, which may also affect biomechanics. The variability in testing speed within and between studies may contribute to inconsistent results regardless of the testing speed methodology, as the variables and observed trends were too inconsistent to establish whether a standardized or preferred speed is a more optimal choice. Rather than statistically controlling for speed [49], testing at multiple standardized speeds may be an optimal solution for future studies.

Most studies evaluated biomechanics under a single speed condition and at baseline (i.e., unexerted state). However, studies incorporating different conditions or states such as an exhausting prolonged run [14, 19, 20, 34], multiple speeds [16, 38], or gait perturbations [21] reported only the change in gait due to these experimental conditions. Reporting only the change in gait, rather than the gait at baseline and all experimental conditions, does not allow for direct comparisons across all studies.

### Kinematics, Kinetics, and Spatiotemporal Variables Using the Traditional Approach

Kinematic, kinetic and spatiotemporal variables were inconsistently associated with running experience given that either no differences in these variables were reported between experience levels [3, 13, 15, 16, 23, 42], or the variables with significant differences were not assessed in more than one study [3, 17, 19, 33, 34, 37, 41, 42, 44, 47].

Traditional approaches were the most common type of biomechanical analysis performed (23/28 studies). Fifteen studies reported discrete, single value variables (e.g., peak joint angle magnitude or knee flexion range of motion). Reducing gait mechanics to discrete variables results in lost data and may misrepresent differences in gait mechanics [50], and thus affect the ability to detect differences between experience groups.

Six studies analyzed kinematics and kinetics variables as a time-series or continuous function to address the limitations of discrete variables [18, 19, 26, 39, 43, 45]. Overall, time-series analyses revealed significant differences in gait variables that were not significantly different when the effect of running experience was explored with discrete variables in the same [19] or different studies [3, 13]. Time-series analysis may be a more sensitive technique to identify gait differences between experience groups because the analysis is performed using all data across the gait cycle. When reporting discrete variables, such as maximum knee flexion or ankle angle at initial contact, all other data from the gait cycle are discarded. It is possible that discrete analyses are not assessing the portions of the gait cycle that differentiate between experience groups. Future research should include both continuous methods of analyses and traditional discrete metrics to uncover differences between running experience groups.

### Systems-Based Analyses

Because traditional analysis methods yielded inconsistent findings, studies adopting a systems-based analysis were conducted to determine whether evaluating the final output of the complex system interactions required for running yielded gait differences between experience groups. Examining motor control strategies and their influence on musculoskeletal loading could enhance the understanding of RRI mechanisms between experience groups [21]. Less-experienced runners exhibited larger long-range correlations for step rate, contact time [21], and stride interval [42], and greater ankle, knee and hip instability evaluated with the Lyapunov exponent [40] compared with more-experienced runners. However, other studies found no differences in long-range correlations between running experience groups for stride interval, spatiotemporal variables, or impact acceleration [14, 17]. These inconsistent results between studies may be due to assessing different yet related biomechanical variables or employing different running protocols (e.g., treadmill versus overground running; exhaustive versus non-exhaustive running). Consequently, it is challenging to draw conclusions regarding the effect of running experience on gait fluctuations.

The relationship of joint and/or segment coordination patterns and variability to running experience was assessed by three studies that employed different methods to quantify coordination (i.e., vector coding and continuous relative phase) and different running protocols [20, 35, 47]. Two studies found lower shank/foot coordination variability among less-experienced runners using vector coding, whereas other segment couplings were not evaluated or significantly different in both studies [20, 35]. A third study using continuous relative phase to evaluate coordination of other couplings found no significant effect of experience [47]. Based on current evidence, it is difficult to conclude if running experience influences coordination patterns or coordination variability because of differences in data collection and analysis methodologies.

### Experience Level in the Context of Running-Related Injuries

Gait differences between more-experienced and less-experienced runners have been proposed to help explain the greater risk of RRI in less-experienced runners [2]. The findings of the present systematic review, however, suggest that there is no significant effect of experience on gait variables given that several studies found no differences between groups and those examining similar variables had inconsistent results. These results do not support the proposed gait-related hypothesis for the differing risk of RRI among more-experienced and less-experienced runners, as described in the reviewed studies. However, this assessment may be premature given the heterogeneity between studies with, for example, the variables investigated, statistical outcome, methodology and procedures, and experience level definitions. Moreover, several recent systematic reviews of prospective RRI studies found only low to moderate evidence that associate gait variables with RRI [6, 10, 51], making the hypothesis that differences in experience-related gait characteristics explain differing RRI risk between groups unwarranted at this time.

There are other potential reasons for a hypothesis linking gait differences between running experience groups to differing RRI risk levels. Observer selection bias may influence RRI rates between more- and less-experienced runners, given that novices who develop RRIs tend to cease running post-RRI [1] whereas those less prone to RRI may go on to become more-experienced runners. Other factors such as previous injury [8, 9, 52, 53], sex [54], tissue specific load capacity [55], menstrual irregularity among women [53, 56], and observer selection bias should be considered in addition to running mechanics when investigating potential RRI risk factors among runners of all experience levels. Ultimately, prospective research encompassing biomechanical, anthropometric, physiological, and training factors of runners of various experience levels is necessary to understand the discrepancy in RRI rates.

### Limitations

This review had the following limitations: (i) A meta-analysis was not possible given that few studies measured the same variable and rarely used similar protocols and definition for the experience groups. (ii) Methodological quality was assessed using the Downs and Black checklist, which was not specifically designed for the evaluation of cross-sectional studies, although it is frequently used in systematic reviews with this design [9, 57, 58]. (iii) All included studies were cross-sectional, which highlights the need for examining longitudinal changes in gait as runners progressed from less- to more-experienced and the influence of experience-related gait differences on prospective RRI development.

### The Challenges in Experience Classification

The primary findings of this review suggest a standard experience group classification method must be established before additional research is performed to elucidate if or how gait biomechanics differs between experience levels. However, selecting the metrics for a running experience classification method is challenging because there is currently no gold-standard motor pattern for running.

The motor learning literature suggests that 10 years or 10,000 h of practice is necessary to be considered an expert in an activity [59–61], albeit most of this research examines learned motor skills and cognitive tasks, such as juggling and typing [61]. In contrast to learned motor skills, running is a fundamental motor skill for which most humans possess the neural network to perform at birth [62, 63]. Running is achieved by a multitude of coordination patterns, for which there is no defined gold-standard motor pattern and so skill or experience is difficult to define [64, 65]. Running performance (i.e., ability to sustain fast endurance running speeds) may be an appealing alternative for defining running skill given its association to competitive outcomes. However, performance is largely dictated by non-biomechanical variables such as maximal oxygen consumption, running economy, and anaerobic threshold [66, 67] rather than a specific motor pattern. Furthermore, these performance-based variables may depend more on incorporating various types of running training than on experience (as defined by the studies included in this review), and have not been linked with spatiotemporal, kinematic, or kinetic factors [68]. The absence of RRI is a possible benchmark for a gold-standard motor pattern, but no clear definition of this gold-standard is available given the lack of consistent prospective evidence for specific biomechanical risk factors of RRI [10].

Given that the absence of a universal definition for a gold-standard running pattern, researchers hold differing perspectives on how to define experience. These differing perspectives, and the potential need to facilitate the recruitment process, likely contribute to the various definitions of running experience used across studies. This heterogeneity makes between-study comparisons difficult. Furthermore, the various definitions could lead to a single participant being defined as a novice in one study but as experienced in another (Table 4). Collectively, these issues hinder the ability to identify gait differences between different running experience levels.

Most studies use the number of years of participation and weekly distance as classification criteria. Table 4 shows that definitions of “experienced” runners (or elite, expert, well-trained, etc.) range between 2–10 years of practice, or a weekly distance between 15–50 km/week. These wide ranges result in runners with the same years of experience being classified as a novice in one study and as elite in another [3, 17], which likely affected the inconsistent results found in this systematic review. Another classifying criterion adopted by only a few studies was the concept of “deliberate practice”, which are activities performed with the sole purpose of improving performance [59]. With this definition, runners who engage in activities to improve technique, speed, and/or endurance would have a different degree of experience than those running for pleasure or who do not perform these activities, yet their years running and weekly distance may be identical.

Roveri et al. [61] proposed a “fuzzy decision support system” analysis to classify participants based on training frequency, training volume, years of practice, and participation in races. However, to date, this approach has been adopted by only one other study [69]. This classification system may be impractical to adopt because criteria (very short, short, moderate, or long) were based on coaches’ subjective opinions, instead of objective numerical cut-offs (e.g., very short: < 1 year, long: > 10 years, etc.).

A more recent classification procedure was proposed by Honert et al. [70] considering the opinion of 142 running footwear experts. That study provided clearly defined cut-offs dividing runners into three experience categories: novice, recreational, and high-caliber. Each category considers aspects of “experience”, “habits”, “performance” and “motivation”, which are all relevant to characterizing a runner. However, considering all of these facets concomitantly can create difficulties categorizing and recruiting participants because it is possible for an individual to be classified in different experience categories for each facet. For example, an individual that started running five years ago to improve general health, runs four times a week and has a 5 km time of 32-min would be considered high-caliber regarding “experience”, recreational regarding “habits” and a novice regarding “performance” and “motivation”. Furthermore, the categories conflate experience with motivation– a high-caliber runner could run for recreational motivations. The same issue of considering multiple facets of experience concomitantly was clearly present in the definitions used in the studies included in this systematic review and likely contributed to the lack of consistency in the results.

Ultimately, the major limitations of the definitions used for each facet across studies are that the definitions are based on cut-offs, or thresholds, which discretizes continuous variables into categorical variables and that these thresholds are inconsistent across studies. Discretizing continuous variables may lead to findings that are not clinically meaningful. This situation may arise when runners are very close to the threshold between more-experienced and less-experienced. Future studies should adopt a continuous approach to associate experience metrics with features of running gait to identify if and when gait changes as a function of experience. Both continuous and categorical approaches for defining running experience have their merit and value, but the ‘correct’ approach depends on the research question.

Treating experience as a categorical variable does have advantages. For example, a group-based approach strengthens the confidence in causal inference between the dependent variable and the outcome. Furthermore, categorization can aid in targeted participant recruitment strategies to ensure the appropriate sample is enrolled to answer the research question. Treating each facet as a categorical variable can facilitate interpretation and comparison, but only if a standard method for classifying runners into experience groups is defined. A standard categorization/discretization approach for running experience allows for studies involving any aspect of running to classify their participants’ experience level, which will enhance the ability to compare results between studies. Methods that allow for improved comparison and consistency between studies may help resolve methodological heterogeneity and allow trends to be observed across the literature that aid in resolving conflicting findings. While a continuous approach is required to determine evidenced-based, non-arbitrary thresholds for defining various facets of experience, until such studies are completed, a standardized definition of experience must be developed and adopted in future research to overcome the influence of varying running experience definitions on the ability to detect gait differences between groups and between studies.

### An Updated Taxonomy for Running Experience

**Fig. 2:**
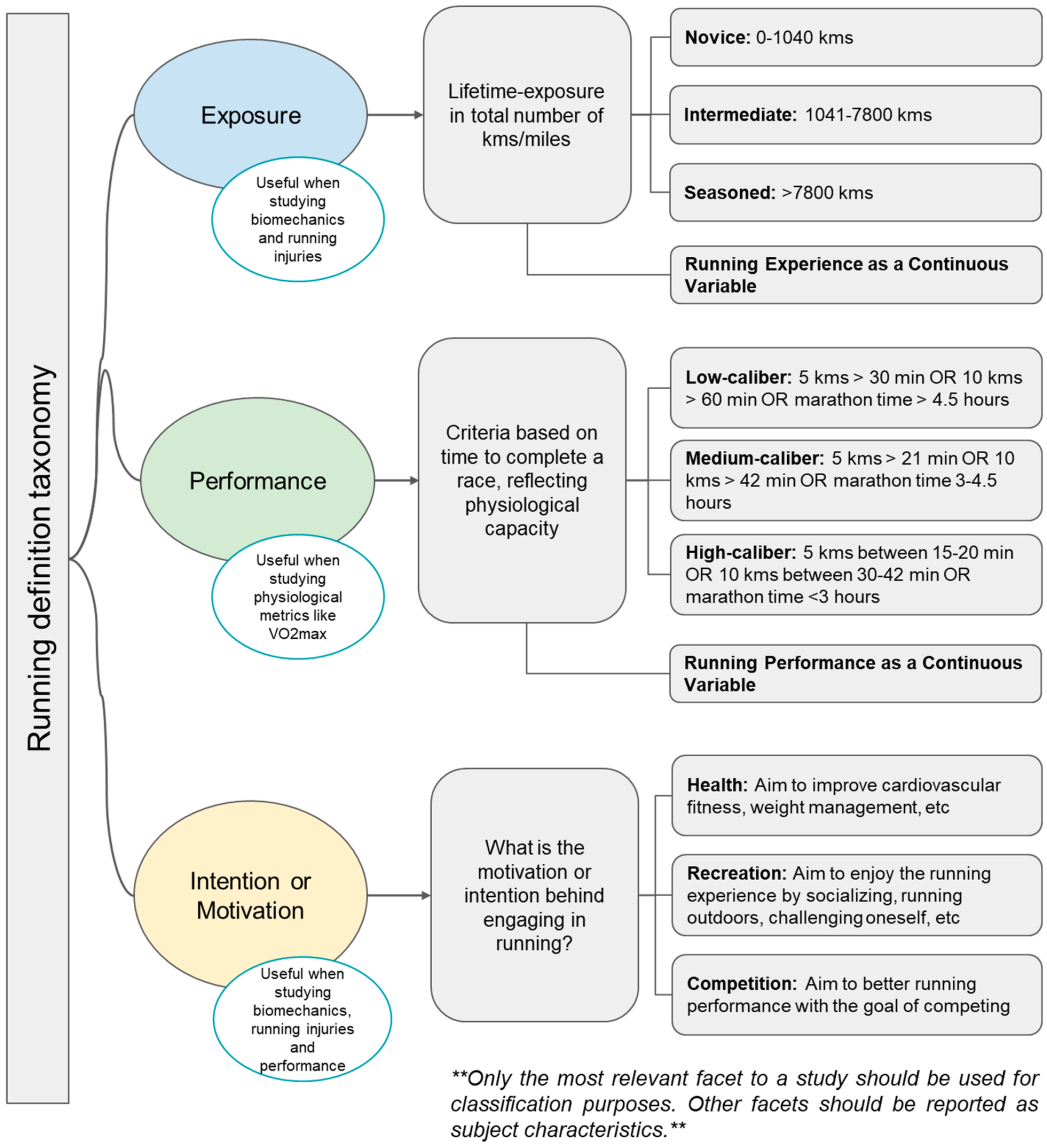
Proposed taxonomy for running experience based on three facets of running exposures.

We propose a taxonomy for running experience which expands on the definitions by Honert et al. [70] that includes three facets: *exposure, performance, and intention*. Each facet has three levels of classification with standardized terminology in accordance with and specific to each facet. Using different terminology for each facet avoids the confusion resulting from using the same terms for different (or combined) components of experience. Unlike Honert et al. [70], we recommend that only one facet deemed most important for the given research question be adopted to classify participants into experience groups, but all three facets should be reported. Examples of such research questions include understanding running training adaptations and questions within the context of RRI, such as the studies included in this systematic review. Using one facet to classify running experience avoids between-facet classification conflicts, as noted in the previous section. Additional facets may be used to classify experience only if it is necessary to answer the research question, but we recommend researchers use variables from the other two facets as covariates, mediators, or moderating variables instead. Facets not used for experience classification should be reported as participant characteristics to allow comparisons between studies and so that the experience level of the included participants as defined by the unused facets can be assessed (Fig. 2). Additional variables that fit within each facet should be measured and accounted for statistically, if appropriate to answer the research question. Each facet is discussed in individual sections below.

#### Exposure

We propose that the running exposure classification facet be defined as lifetime exposure to running quantified as the approximate total number of miles run since the individual first started running. Use of written, electronic logs, or wearable device data is the preferable collection method given the limitations of self-report, especially over many years of running. This facet may be relevant, for example, to studies examining the effects of cumulative loads on running injury risk; Lifetime exposure to running dictates tissue adaptation and morphology, thereby the musculoskeletal tissue stresses experienced and their susceptibility to future RRI. This facet may also be relevant to studies considering the effects of running coordination patterns and variability on RRI risk as running coordination patterns may be influenced by the time spent engaging in the motor-control strategies utilized during running. Specific practice of a novel gait task has been shown to benefit task performance [71], suggesting a benefit of practice on motor control strategies. Treating this exposure facet as a continuous variable will allow for research to identify the exposure magnitude required to elicit changes in gait as running experience progresses. The categorization terminology we propose for this facet is (from lowest to greatest experience): novice, intermediate, and seasoned.

Per Honert et al. [70], exposure to running can be assessed by both running experience (number of years of regular running experience) and running habits (weekly session frequency and distance). Using both criteria separately may present statistical and recruitment challenges, particularly for the categorization approach, as one facet does not over-rule another and categorization would be subjective and potentially differ between researchers.

Given that years and weekly distance measure exposure to running, exposure can be combined into a single value instead of treating running experience and running habits as separate facets. This proposed exposure criterion yields total distance run in the individual’s lifetime which, from a motor learning perspective, may better reflect the influence of running exposure on running gait. An individual running 10 km/week every week for 2-years would have a lifetime running exposure of (10 km/week) * (52 week/year) * (2 years) = 1040 km. These values could be assessed as the running exposure independent variable into a statistical model using the continuous approach. In the categorization approach, we propose an adaptation from Honert et al. [70]. Converting the experience and habits thresholds from Honert et al. [70] into a single value, runners with a lifetime exposure of 0–1040 km would be classified as novice, 780–7800 km as recreational (assuming the lower threshold for high-caliber is the upper threshold for ‘recreational’), and ≥ 7801 km as high-caliber. However, the classification difficulty problem remains for runners with a lifetime exposure of 780–1040 km. In this case, we propose to adopt the upper threshold of the less-exposed group as the lower threshold of the more-exposed group (i.e., novice = 0–1040 km, recreational = 1041–7800 km, high-caliber ≥ 7801 km), but these thresholds have not been validated and/or tested for a consensus.

This proposed running exposure facet, whether assessed as a continuous or categorical variable, addresses problems associated with participants meeting the criteria for multiple groups, yet it does not address exposure metrics such as session frequency (i.e., runs per week), typical single bout duration, distance, and intensity, and significant periods of time off. Run duration, for example, may be an important metric for cumulative load or cumulative damage studies [72], and should be considered as an inclusion/exclusion criteria or additional exposure variable or covariate.

#### Performance

We propose that a performance-based classification be reserved for studies assessing only physiological metrics like maximal oxygen consumption, running economy, or when assessing how gait patterns are related to one’s physiological capacity given that performance can but does not necessarily relate to experience, as described in the "The challenges in experience classification" section. Both a continuous approach and a categorization approach can be used. We propose that participants should be categorized (from lowest to greatest) into low-caliber, medium-caliber, and high-caliber.

The performance-based classification of Honert et al. [70] is defined using thresholds of 5 km, 10 km, and half-marathon finishing times, but requires one slight modification to include the marathon within the lowest performance group; finishing time thresholds for the 5 km and 10 km are the only races included in all performance groups. Excluding the marathon may be reasonable considering the lowest performance group completes runs less than 20 km/week according to another facet in their classification method. However, when considering only the performance facet, requiring no marathon performance or a maximum finishing time of 4.5 h may result in the inability to classify some runners with the Honert et al. [70] method. Therefore, we recommend including a marathon time above 4.5 h as the cut-off for the low-caliber runners and maintain the thresholds for medium-caliber and high-caliber proposed by Honert et al. [70].

When formulating inclusion/exclusion criteria for biomechanics studies, it is important to consider that not all individuals who run may participate in races or be aware of their 5 km, 10 km, or half- or full-marathon times. Excluding those who do not compete or who are not aware of personal best times may unnecessarily present recruitment challenges and limit sample sizes if the research question is not performance-based. For example, the distance or speed —not the time— is important for RRI research questions considering cumulative loading or cumulative damage [72], and so a performance-based metric may not be appropriate for all research questions. In addition, some runners may not be motivated by knowing or improving their personal best, making them a distinct population from those whose aim is to become a faster runner.

#### Intention and Motivation

The third facet we propose addresses motivation and intention with some differences from Honert et al. [70]. Running motivation and intention are important criteria to consider as they may influence susceptibility to RRI [73], potentially by influencing runners’ training choices. For example, someone who runs solely for recreation may choose to forego a running session in favor of another exercise, social, or work-related activity or may not push themselves to increase mileage or intensity in the same way as a competitive runner or a runner determined to improve health. A runner motivated by health or competition may also be more consistent regarding their running training. Such differences in training choices could lead to varied running exposure or likelihood of making a training error that leads to differences in RRI between intention and motivation groups.

In this facet, we diverge from the criteria proposed by Honert et al. [70], that included three possible motivations for each group (with some repeated in multiple groups). Considering multiple motivations simultaneously, which are repeated in different groups and do not have a specific order of importance, gives the classification procedure some uncertainty that may result in lack of standardization in future studies. Therefore, we reduce the possible motivations into three groups: health, recreation, and competition. Although it is possible for a runner to have multiple motivations, only the primary motivation should be used to group runners.

## Conclusions

The current literature is insufficient in volume and homogeneity to identify effects of experience on running biomechanics. Although many gait variables were investigated, most studies found no effects of experience, effects of experience for only one of many variables, or effects of experience that were not confirmed by studies measuring similar variables. Besides the possibility that biomechanics does not change with experience, the lack of consistent results may result from the difficulty of defining the term “experience” and thus the inconsistent criteria by which participants were classified into experience groups. To address these limitations, additional research is necessary to examine the influence of running experience on gait biomechanics that adopt a standard classification definition. Prospective studies that evaluated individuals as they gain running experience would be extremely valuable to observe if changes in biomechanics occur with experience and to assess if those biomechanics influence RRI risk.

## Supplementary Information

Supplementary Material 1.

## Data Availability

All data are available in the manuscript. Information regarding excluded studies is available from the authors by request.

## Abbreviations

RRI: Running-related injuries
Km: Kilometers
PROSPERO: International prospective register of systematic reviews
PRISMA: Preferred reporting items for systematic reviews and meta-analyses 2020
m/s: Meters per second
LRC: Long-range correlations
