# Supplementary material for: Inconsistent Effects of Experience on Running Biomechanics May be Influenced by Study Heterogeneity and Classification Criteria: a Systematic Review and Proposal of a Revised Taxonomy
Source: Sports Med Open. 2025 Jun 8;11:69. doi: 10.1186/s40798-025-00870-5 (PMC12146231; doi:10.1186/s40798-025-00870-5)
Supplement: Supplementary file 1 — Supplementary Material 1. [file 40798_2025_870_MOESM1_ESM.docx]

Inconsistent effects of experience on running biomechanics may be influenced by study heterogeneity and classification criteria: a systematic review and proposal of a revised taxonomy.

*Sports Medicine – Open*

Rodrigo Rabello^1,2,3^, Gauri A Desai^1,4^, Allison H Gruber^1^

^1^ HH Morris Human Performance Laboratories, Department of Kinesiology, School of Public Health–Bloomington, Indiana University, Bloomington, Indiana, USA

^2^ Department of Biomedical Sciences for Health, Università degli Studi di Milano, Milan, Italy

^3^ Sports and Exercise Medicine, Queen Mary University of London, London, UK

^4^ Department of Kinesiology, School of Public Health, University of Maryland College Park, College Park, Maryland, USA

**Supplementary Material 1 –** Search strategy for each database

**PubMed**

((Runn*[Title/Abstract])

AND ((Experience*[Title/Abstract]) OR (Novice[Title/Abstract]) OR (beginner[Title/Abstract]) OR (recreational [Title/Abstract]) OR (competitive [Title/Abstract]) OR (skill* [Title/Abstract]))

AND ((Biomechanic*[Title/Abstract]) OR (kinetic* [Title/Abstract]) OR (spatiotemporal [Title/Abstract]) OR (kinematic* [Title/Abstract]) OR (coordination [Title/Abstract]) OR (stride [Title/Abstract]) OR (step [Title/Abstract])))

**EMBASE**

runn*:ti,ab,kw

AND (experience*:ti,ab,kw OR novice:ti,ab,kw OR beginner:ti,ab,kw OR recreational:ti,ab,kw OR competitive:ti,ab,kw OR skill*:ti,ab,kw)

AND (biomechanic*:ti,ab,kw OR kinetic*:ti,ab,kw OR spatiotemporal:ti,ab,kw OR kinematic*:ti,ab,kw OR coordination:ti,ab,kw OR stride:ti,ab,kw OR step:ti,ab,kw)

**Web of Science**

(TI=(runn*) OR AB=(runn*))

AND (TI=((experience*) OR (novice*) OR (beginner) OR (recreational) OR (competitive) OR (skill*)) OR AB=((experience*) OR (novice*) OR (beginner) OR (recreational) OR (competitive) OR (skill*)))

AND (TI=((biomechanic*) OR (kinetic*) OR (spatiotemporal) OR (kinematic*) OR (coordination) OR (stride) OR (step)) OR AB=((biomechanic*) OR (kinetic*) OR (spatiotemporal) OR (kinematic*) OR (coordination) OR (stride) OR (step)))

**CINAHL and SPORTSDiscus**

(TI runn* OR AB=runn*)

AND (TI experience* OR TI novice* OR TI beginner OR TI recreational OR TI competitive OR TI skill* OR AB experience* OR AB novice* OR AB beginner OR AB recreational OR AB competitive OR AB skill*)

AND (TI biomechanic* OR TI kinetic* OR TI spatiotemporal OR TI kinematic* OR TI coordination OR TI stride OR TI step OR AB biomechanic* OR AB kinetic* OR AB spatiotemporal OR AB kinematic* OR AB coordination OR AB stride OR AB step)
